# Culture, goal orientation and achievement of vocational college students

**DOI:** 10.3389/fpsyg.2025.1639938

**Published:** 2025-08-22

**Authors:** Xuefei Lin, Peiyao Lei, Qi Tan, Bin Xiong

**Affiliations:** ^1^School of Mathematical Sciences, East China Normal University, Shanghai, China; ^2^Shanghai Key Laboratory of Pure Mathematics and Mathematical Practice, Shanghai, China; ^3^Chengdu Academy of Education Sciences, Chengdu, China

**Keywords:** achievement goal orientation, cultural values, mathematics achievement, mathematics motivation, social goal orientation, vocational college students

## Abstract

**Introduction:**

This study explored the relationship between cultural values, goal orientation, and mathematics achievement in mainland China.

**Methods:**

Structural equation modelling was used to analyze data collected from 1,004 first-year students of four majors in higher vocational colleges. This study adopted a four-dimensional goal orientation, including achievement (i.e., students’ mastery, performance-approach, and performance-avoidance) and society (i.e., family instrumental support goal orientation, and family emotional support goal orientation) to describe Chinese students’ motivation.

**Results:**

The results indicated that mathematics achievement was positively related to mastery goal orientations and negatively related to the performance-avoidance goal orientation. Family recognition through achievement was found to positively predict the students’ mastery, performance-approach, performance-avoidance, while conformity to norms was negatively linked to performance-approach orientations. Collectivism, on the other hand, was positively related to adopting the family support goal orientation and to mastery goal orientations, performance-approach. Nevertheless, emotional self-control was positively associated with students’ mastery. Humility was positively related to performance-approach orientations, family instrumental support goal orientation and family emotional support goal orientation. It also had a direct positive predictive effect on mathematics achievement. The results of the bootstrap analyses further confirmed the mediating role of goal orientation in the relationship between cultural values and mathematics achievement: there were positive indirect paths through the mastery orientations, and negative indirect paths through the performance-avoidance goal orientation.

**Discussion:**

Potential explanations for the findings are discussed. This will help reveal the group specificity of cultural values and goal orientation theory, thereby supplementing the contextual adaptability of the theory.

## Introduction

The interest in vocational education has been widely increased in recent years, which is a phenomenon that has been seen not only in China but also in other parts of Asia, such as Indonesia (Ali et al., 2020) and Malaysia (Omar et al., 2011). The growing popularity of vocational education is partly attributed to policy reasons—such as the establishment of the Vocational Education Research Theme by the Chinese Society of Mathematics Education (2024)—and partly the inevitable result of the East Asian cultural context. Influenced by Confucianism, students in East Asian cultural context put greater emphasis on filial piety and hope to pursue excellence than those in other regions. However, Confucianism fostered a discriminatory attitude towards vocational education, with the prevailing view that only students with poor grades end up receiving vocational education (Jing et al., 2022). In reality, it is also true that vocational students are more likely to be at a disadvantage in China’s junior high school to senior high school examinations. Moreover, math holds a relatively low status in vocational institutions, in terms of curriculum standards (Wu and Ye, 2018), because vocational students tend to devote more to technical learning than to math learning. This not only keeps students from aspiring to higher math pursuits, but also alleviates the guilt and negative emotions they feel when failing to meet their parents’ expectations in a high-pressure environment (Tao and Hong, 2014; Wang et al., 2018). However, vocational education (especially higher education) requires adequate attention (Brezavšček et al., 2020). Mathematical achievement is considered a necessary condition not only for academic success but also for effective functioning in daily life (Carey et al., 2017). Strong mathematical learning abilities not only facilitate the development of students’ self-regulated learning (Mejeh and Held, 2022) but also lay the foundation for their future career success (Muhrman, 2022).

Students’ mathematics achievement is influenced by a range of affective factors, among which the most significant one is mathematics motivation (Chen and Lin, 2019; Guo et al., 2021; Zhu and Leung, 2010; Ryan and Deci, 2020). Goal theory is one of the most prominent theories describing achievement motivation (Wolters, 2004). It focuses on the causes and purposes of achievement-related behaviors, including mastery, performance-approach and performance-avoidance orientations (Wolters, 2004). In the Asian region, prior research (Guo et al., 2021) has found that in addition to individually oriented achievement goals, students’ learning behaviors are strongly driven by social factors, particularly family-oriented motivations such as rewarding parental sacrifice, avoiding parental disappointment, and bringing honor to the family (Tao and Hong, 2014; Wang et al., 2018). As a result, some researchers have proposed frameworks that include achievement goals (e.g., GOALS-S) and social goals (e.g., Family-Support Goals and Social Goals Framework) (Dowson and McInerney, 2004; King and McInerney, 2019) to describe Asian students’ motivations. It is hypothesized that the goal orientations of students in this study include both achievement goal orientations and social goal orientations.

### Research questions

RQ1: What is the relationship among cultural values, goal orientation, and math achievement for students in Chinese vocational institutions?

RQ2: What are the similarities and differences between such a relationship and Chinese elementary and middle school students in a highly competitive situation?

## Literature review

### Conceptualization of culture

Culture is an abstract concept that has been interpreted in various ways by scholars. For example, Pinxten (1995) explores the conceptualization of time in Navajo Indian culture, emphasizing the cultural understandings of time as a complex phenomenon rather than a natural one, which highlights the significance of studying time as a cultural construct. Barnett (2001) discusses Foucault’s approach to culture as an object of analysis, focusing on the relationships between culture and power. This perspective offers a usable definition of culture that avoids abstract and generalizing notions, providing a distinct conceptualization of culture in relation to governance. Furthermore, there are also many researchers who have given methods of measuring this dimension of culture. Hofstede and Bond (1988) put forward a typology with five dimensions: power distance, uncertainty avoidance, individualism versus collectivism, masculinity versus femininity, and long-term versus short-term orientation. Leung et al. (2009) proposed a framework containing 10 values: benevolence, universalism, self-orientation, stimulation, hedonism, achievement, power, security, conformity, and tradition. However, these concepts are overly broad and are not able to measure Asian cultures accurately, especially societies and cultures under the influence of Confucianism.

Kim et al. (2005) proposed a five-dimensional framework to represent important Asian cultural values, including family recognition through achievement, emotional self-control, humility, conformity to norms, and collectivism. It is worth noting that while these values are not unique to Asians, they are particularly salient in the Asian context, and Kim et al. (1999) found that Asian Americans were more inclined to adhere to these values than European Americans. Family recognition through achievement implies that an individual’s academic performance and professional achievements are related to the family’s reputation. Emotional self-control implies that people need to control and conceal their true emotions and behave decently. Humility implies people’s tendency to downplay their successes and avoid talking publicly about their achievements. Norm compliance signifies that people are supposed to fulfil parental and social expectations and abide by family and social norms. Finally, collectivism means that people should prioritize the needs of the collective over the needs of the individual.

The multidimensional cultural values framework proposed by Kim et al. (2005) was adopted in this study because it has a high degree of cultural compatibility with Chinese culture. It can reflect salient cultural factors in the Asian context, and this framework has been practically validated in the Chinese socio-cultural context (Guo et al., 2021).

### Goal orientation theory

This study defines mathematical goal orientation as the purpose and reason for motivating students to strive to learn mathematics. Early research identified two types of goal orientation: mastery and performance (Ames, 1992). While proficient goal-oriented students seek to understand mathematics and improve their abilities, performance goal-oriented ones emphasize outperforming their peers in mathematics. Recent research has classified performance goal orientations into two types: performance approach and performance avoidance to distinguish their different roles in student learning (Elliot and Church, 1997). Students with performance-approach goal orientations emphasize demonstrating mathematical competence and performing better than others (Dweck, 2006, pp. 189–195) by working hard, whereas students with performance-avoidance goal orientations focus on avoiding mathematical failure or being perceived as incompetent (Oberhauser and Hertel, 2023). Recently, researchers have further categorized mastery-orientation as approach and avoidance (Elliot and McGregor, 2001). However, mastery-avoidance orientations were not included in this study, because the three-part model (i.e., mastery, performance approach, and performance avoidance orientations) is the most widely used achievement goal framework in recent years in research (Chen, 2015; Chen and Wong, 2015; Elliot, 2006; Levontin and Bardi, 2019).

#### Family goal orientation

In collectivist societies, students’ social goals are closely related to their families. Students with family support goals cite supporting and helping family members as motivations for working hard in school. King and McInerney (2019) found that family support goals were the most frequently reported social goals of Filipino students. Family and parent-related motivations shape students’ achievement behaviors in different regions of China, such as Beijing (Cheung and Pomerantz, 2012), Hong Kong (Chan and Lai, 2008; Tao and Hong, 2014), etc. According to Farmer and Farmer (1996), family support is generally categorized into emotional and instrumental support, yet the two types of support are often conflated in reporting. This phenomenon is particularly prevalent when reporting family support goals. For instance, King and McInerney (2019), and Guo et al. (2021) both adopt a goal-oriented instrumental aggregation perspective when reporting support orientation. Single-issue studies have also been used. For example, Xu and Jin (2024) examined emotional support. For family instrumental support goals, take rural Jiangxi (Wang et al., 2018) as an example, parental expectations are students obtaining a higher degree. The prominent reason for Korean students to engage in learning is to support their families (Lee and Bong, 2016). For family emotional support goal, my parents can be proud of me (Wills and Cleary, 1996). Although there is a large degree of overlap between the two, this study seeks to differentiate between these two support goals in order to explore the impact of both instrumental and emotional dimensions on goal orientation.

### Relationship among cultural values, goal orientations, and mathematics achievement

The impact of cultural values on students’ motivation and academic performance, particularly in mathematics, has been a subject of significant interest among researchers. Studies have consistently indicated that these values play a pivotal role in shaping students’ goal orientations (Hau and Ho, 2008; King and McInerney, 2019; Levontin and Bardi, 2019; Liem et al., 2011; Liem and Nie, 2008; Chen, 2015) and their performance in mathematics (Leung, 2014). For example, Leung (2014) highlighted that the mathematics learning process and outcomes for East Asian students are often influenced by Confucian values, including high educational expectations, an emphasis on examinations, a belief in the importance of effort, and humility.

This study seeks to delve deeper into the manner in which prominent cultural values in Chinese societies—such as collectivism and humility—impact students’ mathematics goal orientation and performance. Drawing on the cultural values framework (Kim et al., 2005), goal orientation theory (Elliot and Church, 1997; Jiang et al., 2019), and supported by inferences and findings from prior research (Hau and Ho, 2008; King and McInerney, 2019; Levontin and Bardi, 2019; Liem et al., 2011; Liem and Nie, 2008; Xu and Jin, 2024), this section discusses the anticipated interplay among cultural values, goal orientations, and mathematics performance.

### Basic hypothesis

#### Cultural values and goal orientations

Collectivism is a prominent element of Asian culture (Hau and Ho, 2008; Leung, 2021). Researchers have found that, influenced by the culture of collectivism, Chinese students work hard in school not only for themselves but also for their families (Tao and Hong, 2014). The family’s future and interests are closely related to children’s academic performance (Tao and Hong, 2014) in China (Hypothesis 1).

Liem and Nie (2008) investigated the relationship between cultural values and achievement motivation among secondary school students in China and Indonesia. They found that conformity to norms positively predicted students’ mastery and performance-approach orientations. Conformity to norms is positively related to students’ mastery (Hypothesis 2a), performance approach (Hypothesis 2b) in mathematics learning.

In Chinese culture, family recognition through achievement is closely related to the concept of family reputation (Han, 2016; Hwang, 2006). A student’s academic achievement can bring honor or shame to the entire family (Hwang, 2006). Family recognition through achievement is positively related to performance approach (Hypothesis 3a), performance avoidance (Hypothesis 3b) and family support orientation (Hypothesis 3c).

Emotional self-control is a universal value in the Chinese cultural context. Luo et al. (2013) points out that Chinese people are expected to control their emotions and behave appropriately, which helps students adapt to society. However, self-restraint may lead students to sacrifice their preferences and suppress their feelings in order to meet others’ needs. Similarly, in Guo et al. (2021) study, it was noted that emotional self-control had positive predictive effect among fifth-grade students who were more inclined to avoid failure. This leaves the effect of emotional self-control on math learning not explicitly stated. We still accept the original hypothesis (Hypothesis 4) that emotional self-control can have a positive impact on performance avoidance.

Humility is a traditional virtue in Confucian culture. However, existing studies have yet to reach a conclusion that humility has a significant influence in Confucian culture. Not only that, in Guo et al. (2021) study there was a link between humility and any goal orientation and math achievement, which makes it necessary to take a cautious approach towards humility in the inquiry (Hypothesis 5).

#### Goal orientations and mathematics achievements

The important role of goal orientation in shaping student achievement in mathematics is well established (Keys et al., 2012; Mouratidis et al., 2018; Wolters, 2004). Research has shown that mastery orientations have a positive impact on student engagement and mathematics achievement (Keys et al., 2012; Wolters, 2004; Guo et al., 2021) (Hypothesis 6).

Researchers examined the impact of family support goal orientation on student learning outcomes (King and McInerney, 2019). In a survey of 1,101 Filipino secondary school students, King and McInerney (2019) found that family support goals were positively associated with students’ academic engagement and achievement. Since we have refined family goal orientation into family instrumental support orientation and family emotional support orientation, we hypothesize that family support goal orientation could positively predict students’ mathematics achievement (Hypothesis 7).

All hypotheses are summarized as follows:

*Hypothesis 1:* Collectivism is positively related to family support orientation.

*Hypothesis 2a:* Conformity to norms is positively related to students’ mastery in mathematics learning.

*Hypothesis 2b:* Conformity to norms is positively related to performance approach.

*Hypothesis 3a:* Family recognition through achievement is positively related to performance approach.

*Hypothesis 3b:* Family recognition through achievement is positively related to performance avoidance.

*Hypothesis 3c:* Family recognition through achievement is positively related to family support orientation.

*Hypothesis 4:* Emotional self-control is positively related to performance avoidance.

*Hypothesis 5:* Humility is positively related to mathematics achievement.

*Hypothesis 6:* Students’ mastery can boost student achievement in mathematics.

*Hypothesis 7:* Both family instrumental support orientation and family emotional support orientation could positively predict students’ mathematics achievement.

### The mediating role of goal orientation between cultural values and mathematics achievement

Although plentiful studies have highlighted the close relationship between cultural values and goal orientation (Hau and Ho, 2008; King and McInerney, 2019; Liem et al., 2011; Liem and Nie, 2008; Zusho and Clayton, 2011) as well as the close relationship between goal orientation and mathematics achievement (Keys et al., 2012; Mouratidis et al., 2018; Wolters, 2004), few studies have examined the mediating role of goal orientation between cultural values and mathematics achievement. Liem et al. (2011) are the only ones who have examined the relationship between student values, achievement motivation, and academic achievement. In addition, Guo et al. (2021) explored the relationship between student values and academic achievement at the primary school level. According to Guo’s findings, cultural factors such as family support positively contribute to mathematics achievement in East Asian cultures, but the authors themselves acknowledge that this finding has little application due to factors such as the sample.

In order to continue exploring the issue of the East Asian Cultural Circle and to supplement the sample of Guo et al. (2021), it is expected that an even larger and more universal sample will be used to explain and illustrate this issue. Not only that, there is a need to localize the dimension of humility that it measures, and to make localized changes to the questions that are less credible, in order to expect the questions to be more colloquial and highly credible. Family goal orientation was refined to measure both instrumental and affective dimensions to determine which aspect of the factor has an impact on achievement.

### Vocational school student population

Vocational institutions are not part of the mainstream in China’s Confucian culture. According to Jing et al. (2022) for the student population of vocational institutions in China, students have a relative low status in the public and parents do not want their children to pursue vocational and technical education. Chen (2021) states more bluntly that vocational education is a dead end in the eyes of Chinese parents. It can be seen that this group is disadvantaged in the context of Confucian culture. In addition, the lack of curriculum standards (Wu and Ye, 2018) in vocational education and the fact that mathematics is a marginal subject in vocational education that is not valued by students make the competitive pressure on students to achieve in mathematics almost non-existent, and parents’ pressure on students to take exams and avoidance due to the pressure of collectivism and Confucianism can be considered to be reduced to a minimum (Leung, 2014; Tao and Hong, 2014). This makes it possible to investigate the most realistic cultural values and goal orientations without peer competition. At the same time, mathematics achievement is considered a prerequisite for academic success (Carey et al., 2017) and can also lay the foundation for future career success (Muhrman, 2022), which in turn requires vocational students to devote time and effort to mathematics.

### Theoretical framework

**Figure 1:**
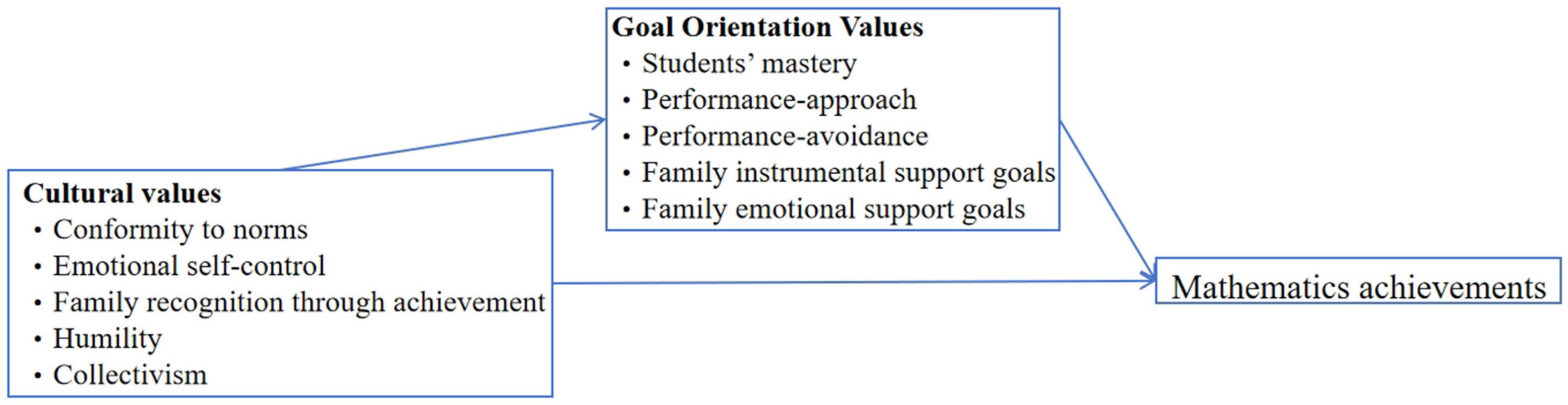
Conceptual framework.

The main purpose of this study is to examine the relationship between cultural values, vocational students’ goal orientation and achievement in the Chinese vocational context. The primary framework of this article is based on Guo et al. (2021) framework in his study of culture and mathematics achievement of primary school students in Guizhou. Kim et al. (2005) proposed an appropriate framework to describe salient values in the Asian cultural context, including family recognition through achievement, emotional self-control, humility, conformity to norms, and collectivism. Based on goal orientation theory (Elliot and Church, 1997; Kaplan and Maehr, 2007; Wills and Cleary, 1996) and goal research in Asian contexts (King and McInerney, 2019), this study adopted a five-dimensional goal orientation (i.e., student’s mastery, performance-approach, and performance avoidance) and social (i.e., family instrumental support goals, and family emotional support goals)—to describe Chinese students’ motivation. As noted earlier, previous researchers have found a strong relationship between cultural values and goal orientation (Levontin and Bardi, 2019; Liem and Nie, 2008) as well as between goal orientation and mathematics achievement (Keys et al., 2012; Mouratidis et al., 2018; Wolters, 2004). In addition, Liem et al. (2013) confirmed the mediating role of goal orientation between cultural values and mathematics achievement. Based on these findings, this study hypothesizes 1–7 as mentioned above that Chinese students’ cultural values are related to their goal orientation and further related to their mathematics achievement. The conceptual framework of this study is shown in Figure 1.

## Methodology

### Ethics statement

This study obtained informed consent from participants and approval from the ethics committee of the authors’ institution.

### Participants and procedures

A total of 1,004 students (male = 887; female = 117) from four faculties of a higher vocational and technical school enrolled in the first year participated in this study, and their mean age was 19.58 years (SD = 0.975). This vocational school enrolls over 8,000 students annually and they are divided into seven faculties with 27 majors. First-year students from 12 majors under four faculties were randomly selected for the survey.

The authors selected the study population using convenience sampling method with the help of local teachers. The students were predominantly Han Chinese with a very small proportion of Manchu and Korean. The case school is a well-known vocational and technical school in Liaoning Province, whose teaching quality and students’ academic performance are at the top of the industry in the region. It’s graduates have a nearly 100 percent employment rate. The average socio-economic status of the students’ families is at the median level in Liaoning Province. The proportion of households with at least one person with a university degree or higher is 10%; the proportion with the highest level of education at tertiary level is 20%; the proportion with the highest level of education at upper secondary level or equivalent is 31%; and the proportion with the highest level of education at lower secondary level is 39%. It should be noted, in particular, that in the item of educational attainment, this sample is basically in line with the true results of the Seventh National Population Census of the People’s Republic of China, indicating a highly representativeness (National Bureau of Statistics of the People's Republic of China, 2021).

This study employed a questionnaire to measure students’ cultural values and mathematical goal orientation, and a five-point Likert scale (ranging from “completely disagree” to “completely agree”) to assess their agreement with these items.

The questionnaire has been ethically vetted to ensure that it does not involve personal privacy. It consists of a total of 40 questions, with the first 10 questions focusing on basic individual information, which contains some basic variables to control. Students’ gender (boy = 1; girl = 2), age (where data on minors were deleted), ethnicity, parents’ highest level of education (junior high school and below = 1; high school or secondary school = 2; college = 3; university and above = 4), and household resources and book ownership (i.e., number of books in the home) were included in the analyses as covariates, where book ownership, due to the fact that modern paper-lessness is relatively widespread, the number of e-books is also included as the number of books to control for their effect on motivation and achievement (Hu et al., 2018a,b; Mohammadpour and Shekarchizadeh, 2013; Mouratidis et al., 2018). Household resources can represent the economic status of the household: controlling using the degree of electronic, having a private computer, separate bedroom, whether the household owns a small car, etc. Here personal mobile phones are not taken into account because the way to fill out the questionnaire was spread using mobile phone Wechat groups, which are filled out using the students’ personal mobile phones. Although teachers at the vocational school helped notify all students, the questionnaire was filled out on a voluntary basis, meaning that data collection could not cover all students from the four faculties. The detailed information is shown in Table 1 below.

**Table 1:** Demographic data of the sample (*N* = 1,004).

Demographic	Type	Frequency	Percentage
Gender	Male	887	88.30%
	Female	117	11.70%
	16	1	0.10%
	17	4	0.40%
	18	121	12.10%
	19	345	34.40%
	20	381	37.90%
	21	136	13.50%
	22	10	1%
	23	4	0.40%
	24	1	0.10%
	25	1	0.10%
Parents’ highest education level	Middle school	396	39.40%
	High school or middle vocational school	307	30.60%
	High vocational school (Associate Degree)	197	19.60%
	Bachelor or Higher	104	10.40%
Books	Less than 10	204	20.30%
	10–50	361	36%
	50–100	178	17.70%
	over 100	261	26%

### Measures

All items on the scale use a standard five-point Likert scale (ranging from “completely disagree” to “completely agree”), unless otherwise specified. And Cronbach alpha was estimated for each group.

#### Cultural values

The second part focuses on cultural values, which were assessed using a 15-item scale adapted from the Asian Values Scale (Kim et al., 1999) and the Asian American Values Scale-Multidimensional (Kim et al., 2005). These scales were selected because they cover salient cultural values in the Chinese context (e.g., humility, conformity to norms, collectivism), which may be important factors in shaping Chinese students’ goal orientation and mathematics achievement (see the literature review section for more information). It is worth noting that these scales were originally developed based on Asian college students in an American context. Some of the entries were selected and modified in order to adapt the scales to Chinese vocational college students. This modification process was carried out in two stages. In the first stage, we directly cited Guo et al. (2021) already localized questionnaire (e.g., emotional self-control, collectivism, etc.). In the second stage, given that Guo et al. (2021) found the reliability and validity of the measurement of the dimension of humility in cultural values to be relatively low, we analyzed some of the possibilities provided and adapted the questionnaire based on the basic circumstances of vocational college students. For example, based on interviews with local teachers, we found that students’ language proficiency was at a relatively low level, so we adapted the questionnaire by modifying some obscure phrases. For example, the phrase “One should have sufficient inner resources to resolve emotional problems” was revised to “People need to be strong inside to control their impulses and not go to the top (This is a Chinese expression that largely maintains the original meaning of the questionnaire).” To enhance the credibility of the revisions, 10 students from non-sampled majors were randomly selected by the school’s teachers to evaluate the revised questionnaire and engage in private conversations with the researchers to confirm their understanding of the sentences, and the result showed that the 10 students were able to accurately understand the meaning of the questions. It can be concluded that this localization adaptation has a high degree of credibility.

The questionnaire included: collectivism (COM;3 items; α = 0.868); conformity to norms (NORM;3 items; α = 0.901); emotional self-control (SELF;3 items; α = 0.866); family recognition through achievement (FRA;3 items; α = 0.857); humility (HUM;3 items; α = 0.926); culture (15items; α = 0.946). The validation factor analysis was designed to test the validity of the five-factor structure of cultural values, χ^2^(df) = 218.395(80), *p* < 0.001, χ^2^/df = 2.730, RMSEA = 0.042, GFI = 0.971, RFI = 0.977, CFI = 0.985, IFI = 0.989, TLI = 0.985 and indicate an acceptable model fit.

#### Goal orientation values

The third section is the Goal Orientation Scale (Midgley et al., 1998) and Family Support Scale (Wills and Cleary, 1996). This study adapted three sets of items from the Goal Orientation Scale to assess students’ mastery (e.g., An important reason why I do my math work is that I like to learn new things.), performance-approach (e.g., I want to do better than other students in my class.), and performance-avoidance (e.g., It’s very important to me that I do not look stupid in my math classes.).

Similarly, appropriate adaptations were made in the Family Support Scale to suit students in vocational colleges. Although a good deal of the literature (Guo et al., 2021; Jiang et al., 2019; King and McInerney, 2019) suggests uniform reporting on this issue of family support goals, the family instrumental and emotional support goals are generally aggregated uniformly as family support goals. In order to further refine the family support goals, Wills and Cleary's (1996) family support questionnaire was used as the basis for the selection of questions using Xu and Jin (2024) for the Chinese family support goals, and three questions in each of the family emotional support goals (e.g., I’m trying to study maths to satisfy my parents’ spiritual needs.) and the family instrumental support goals (e.g., I’m trying to learn maths to help my parents live a carefree life in the future.) were used to measure the students’ family support goals.

The questionnaire was completed as follows: students’ mastery (MAS;3 items;α = 0.920); performance-approach (PAP;3 items;α = 0.896); performance-avoidance (PAV;3 items;α = 0.863); family instrumental support goal (FS;3 items; α = 0.965); family emotion support goal (FE;3 items;α = 0.957); the hole goal orientation and family support scale (15items; α = 0.875). The validation factor analysis was designed to test the validity of the five-factor structure of cultural values, χ^2^(df) = 209.635(80), *p* < 0.001, χ^2^/df = 2.730, RMSEA = 0.040, GFI = 0.973, RFI = 0.982, CFI = 0.991, IFI = 0.991, TLI = 0.989 and indicate an acceptable model fit. As the two concepts of family instrumental support goal and family emotional support goal were used for the first time in this study, their validity is presented in Table 2 below. Although the two concepts overlap to a great extent, they can still be considered as two distinct conceptual determinations.

**Table 2:** Validity testing of differences in various dimensions of goal orientation scale.

Variables	MAS	PAP	PAV	FS	FE
MAS	0.793				
PAP	0.791	0.744			
PAV	−0.464	−0.399	0.685		
FS	0.572	0.658	−0.338	0.903	
FE	0.585	0.667	−0.319	0.809	0.883
Sqrt of AVE	0.891	0.863	0.828	0.950	0.940

#### Mathematics achievement

Students’ math scores from the Spring Semester 2024 final exam were used as a determination of mathematics achievement on a scale of 0 to 100, with a passing score of 60. The test consisted of simple calculus and linear algebra. It is worth mentioning that the test papers for this exam were commissioned by the school and supervised by the local education bureau. The question-setting panel consisted mainly of experienced mathematics experts, ensuring that the test papers for this exam comply with the mathematics curriculum standards for vocational colleges and that the results are reasonably convincing.

#### Control variables

Gender of student (male = 1; female = 2), highest level of parental education (middle school or less = 1; high school or middle vocational school = 2; high vocational school (associate degree) = 3; bachelor or higher = 4), family resources and book ownership (i.e., the number of books in the home) were used as covariates in the analyses to control for their effects on academic motivation and achievement. Family resources, which represent the economic status of the family, are assessed by the number of items in the home, such as the student’s own room, a study, a computer, a television, and a car.

#### Test of common method bias

The Harman single-factor method was used to test the common method bias of the data. Through unrotated factor analysis of the data, the variance explained by the first factor was found to be 49.397%, which is less than the reference value of 50% (Podsakoff and Organ, 1986). Therefore, the common method bias in this study is within an acceptable range.

### Data analysis

Firstly, the questionnaire was created in a mandatory form so that the questionnaires can be successfully collected with no missing values. Secondly, the questionnaire was set up preventing the same value to be filled throughout in order to enhance the authenticity of the answers.

We used Structural Equation Modelling (SEM) and maximum likelihood estimation of AMOS to examine the relationship between cultural values, goal orientation and mathematics achievement. In this study, χ^2^/df < 5, RMSEA = 0.08_,_ GFI = 0.90, RFI = 0.90, CFI = 0.90, TLI = 0.90 (Kenny and McCoach, 2003) indicated an acceptable model fit. Finally, a bootstrap analysis with 5,000 replications (Hayes, 2009) was conducted to test the mediating role of goal orientation in the relationship between cultural values and student achievement in mathematics. Bootstrap 95% confidence intervals (CIs) not containing 0 indicate a significant mediating effect.

## Results

### Structural equation model

**Figure 2:**
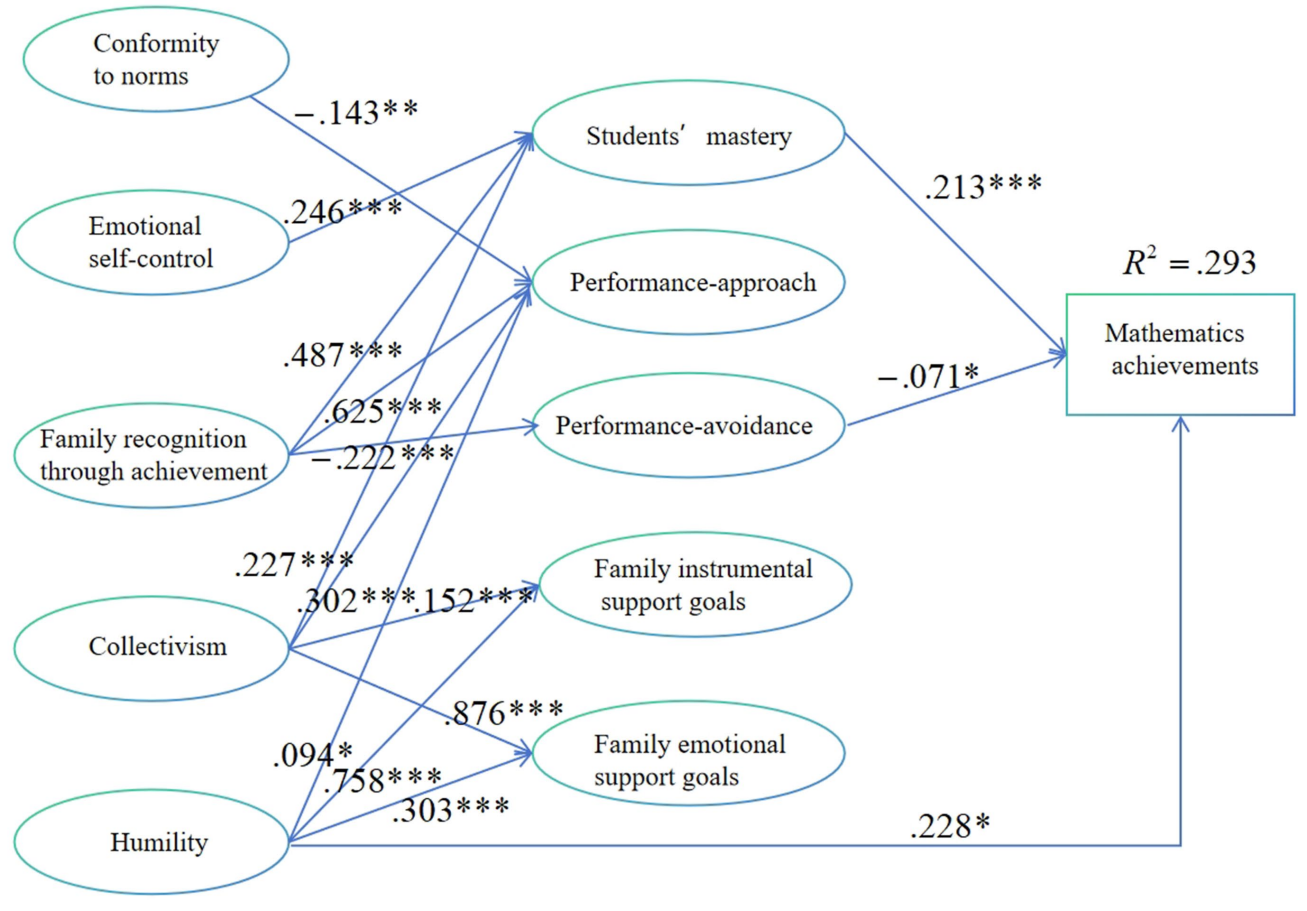
Significant paths in SEM (*p < 0.05, **p < 0.01, ***p < 0.001).

The SEM results showed that our hypothesized model fitted the data well: χ^2^/df = 3.229, RMSEA = 0.047, GFI = 0.923, RFI = 0.952, IFI = 0.972, TLI = 0.966, CFI = 0.972. The significant paths in the model are presented in Figure 2. Our results indicated that mathematics achievement was positively associated with mastery (*β* = 0.213, *p* < 0.001) and humility (*β* = 0.228, *p* = 0.015), but negatively related to performance-avoidance orientation (*β* = 0.071, *p* = 0.031). No significant relationship was found among performance-approach orientation (*β* = 0.05, *p* = 0.462), family instrumental support goal (*β* = 0.031, *p* = 0.696) and family emotional support goal (*β* = 0.085, *p* = 0.24), and some direct effect dimensions collectivism (*β* = 0.051, *p* = 0.557), conformity to norms (*β* = 0.02, *p* = 0.752), emotional self-control (*β* = 0.131, *p* = 0.071); family recognition through achievement (*β* = 0.014, *p* < 0.875).

Family recognition through achievement was positively linked to all the goal orientation dimensions: mastery (*β* = 0.487, *p* < 0.001), performance-approach orientation (*β* = 0.625, *p* < 0.001), performance-avoidance orientation (*β* = 0.222, *p* < 0.001). Collectivism establishes significant associations with almost all goal variables: mastery (*β* = 0.227, *p* < 0.001), performance-approach orientation (*β* = 0.302, *p* < 0.001), family instrumental support goal (*β* = 0.152, *p* < 0.001) and family emotional support goal (*β* = 0.876, *p* < 0.001), but no significant association with performance-avoidance orientation (*β* = 0.058, *p* < 0.402). Humility establishes associations with performance-approach orientation (*β* = 0.094, *p* < 0.044), family instrumental support goal (*β* = 0.758, *p* < 0.001) and family emotional support goal (*β* = 0.303, *p* < 0.001), no significant associations with mastery (*β* = 0.047, *p* < 0.293), performance-avoidance orientation (*β* = 0.07, *p* < 0.286). Emotional self-control only established a link with mastery (*β* = 0.246, *p* < 0.001) and had no significant relationship with any of the remaining variables: performance-approach orientation (*β* = 0.059, *p* < 0.301), performance-avoidance orientation (*β* = 0.148, *p* < 0.063), family instrumental support goal (*β* = 0.007, *p* < 0.863) and family emotional support goal (*β* = 0.028, *p* < 0.526). A similar situation is that of conformity to norms, which a link is established only with performance-approach orientation (*β* = 0.143, *p* < 0.003) and no relationship with other variables: mastery (*β* = 0.019, *p* < 0.685), performance-avoidance orientation (*β* = –0.045, *p* < 0.506), family instrumental support goal (*β* = 0.01, *p* < 0.776) and family emotional support goal (*β* = 0.068, *p* < 0.065).

All the significant paths are shown in Figure 2 below.

### Mediation test

We conducted bootstrap analysis with 5,000 random samples to further explore the mediating role of goal orientation between cultural values and mathematics performance. The results revealed that family recognition through achievement had a positive indirect relation with mathematics achievement via mastery, via performance-approach orientation and had a negative indirect relation via performance-avoidance orientation. Collectivism had a positive indirect relation with mathematics achievement via mastery, via performance-approach orientation, via family instrumental support goal and family emotional support goal. A positive indirect relation with humility and mathematics achievement was found. In goal orientation, mastery, performance-avoidance orientation have a direct connection with math achievement. There is no path via a family support goal, whether it is a family instrumental support goal or a family emotional support goal. The specific situation is shown in Table 3 below.

**Table 3:** Mediation effect test of bootstrap 5,000 (95%).

Parameter	Indirect effects	95% confidence intervals
MAS <−-- FRA	0.130	[0.287, 0.785]
PAP <−-- FRA	0.151	[0.410, 0.973]
PAV <−-- FRA	0.105	[−0.439, −0.022]
FE <−-- HUM	0.061	[0.190, 0.433]
MAS <−-- COM	0.075	[0.077, 0.371]
PAP <−-- COM	0.081	[0.141, 0.459]
FS <−-- COM	0.061	[0.041, 0.278]
FE <−-- COM	0.068	[0.475, 0.750]
Achievement <−-- HUM	0.054	[0.122, 0.337]
Achievement <−-- MAS	0.063	[0.093, 0.336]
Achievement <−-- PAV	0.032	[−0.138, −0.012]

Thus, based on Figure 2 and Table 3, all hypotheses can be verified, as shown in Table 4 below.

**Table 4:** Testing of hypotheses.

Relationships		Path coefficients	*p-*values	Significance (*p* < 0.05)
H1: Collectivism to family instrumental support		0.152	<0.001	Yes
H1: Collectivism to family emotional support		0.626	<0.001	Yes
H2a: Conformity to norms to students’ mastery		0.227	0.685	No
H2b: Conformity to norms to performance approach		–0.143	0.003	Yes
H3a: Family recognition through achievement to performance approach.		0.625	<0.001	Yes
H3b: Family recognition through achievement to performance avoidance.		–0.222	0.001	Yes
H3c: Family recognition through achievement to family support orientation.	Instrumental	0.023	0.492	No
	Emotional	–0.03	0.402	
H4: Emotional self-control to performance avoidance.		–0.148	0.063	No
H5: Humility to mathematics achievement.		0.228	0.015	Yes
H6: Students’ mastery to math achievement.		0.213	0.001	Yes
H7: Family support orientation to mathematics achievement.	Instrumental	–0.031	0.696	No
	Emotional	0.085	0.24	

### Control variables analysis

#### Gender

The gender effect on the model was explored through a multi-cluster analysis, the results of which are shown in Table 5 below. As can be seen, the models remain stable regardless of whether they are unconstrained model, measurement weight model, or structural covariances model. Further results indicate that gender has no significant effect on model construction, but has a noticeable effect on pathways (Δ*χ*^2^/df = 2.27, *p* < 0.001). It can be assumed that gender modulates the path weights of the model.

**Table 5:** Gender impact on modelling.

Model	CMIN	DF	CMIN/DF	RSME	RFI	IFI	TLI	CFI
Unconstrained	1873.446	780	2.402	0.037	0.931	0.965	0.958	0.965
Measurement weights	1910.268	810	2.358	0.037	0.932	0.965	0.96	0.965
Structural weights	1966.913	835	2.356	0.037	0.932	0.964	0.96	0.964
Structural covariances	1995.248	850	2.347	0.037	0.932	0.963	0.96	0.963
Structural residuals	1999.395	855	2.338	0.037	0.932	0.963	0.96	0.963

For male, the following paths were significant: emotional self-control established a link with mastery (*β* = 0.269, *p* < 0.001). Family recognition through achievement linked performance-avoidance orientation (*β* = 0.227, *p* < 0.003). Humility established associations with performance-approach orientation (*β* = 0.1, *p* < 0.04), with family emotional support goal (*β* = 0.331, *p* < 0.001). Collectivism established significant associations with performance-approach orientation (*β* = 0.301, *p* < 0.001). Similarly, math achievement was significant associated with humility (*β* = 0.219, *p* < 0.034), mastery (*β* = 0.203, *p* < 0.002), and performance-avoidance orientation (*β* = 0.08, *p* < 0.023). However, none of these paths are significant for female.

For female, the following paths were significant: conformity to norms established a link with mastery (*β* = 0.398, *p* < 0.005), family instrumental support goal (*β* = 0.271, *p* < 0.019), and family emotional support goal (*β* = 0.395, *p* < 0.001). Emotional self-control showed a negative correlation with family support goals, with family instrumental support goals (*β* = 0.525, *p* < 0.001), and family emotional support goals (*β* = 0.62, *p* < 0.001). Family recognition through achievement linked family instrumental support goals (*β* = 0.291, *p* < 0.001).

#### Highest level of parental education

The unconstrained model runs good (χ^2^/df = 2.572, RMSEA = 0.04, IFI = 0.915, TLI = 0.911, CFI = 0.915, *p* = 0.127) and the Measurement weights path weights change (χ^2^/df = 2.551, RMSEA = 0.039, IFI = 0.915, TLI = 0.912, CFI = 0.915, *p* = 0.001). Interestingly, these weighting changes are all due to the second group (high school or middle vocational school). For example, on the path from emotional self-control to mastery, the second group (*β* = 0.473, *p* < 0.022) was weighted higher than the other groups (*β* = 0.221, *p* < 0.001).

#### Family resources and books ownership

The unconstrained model runs good (χ^2^/df = 2.573, RMSEA = 0.04, IFI = 0.914, TLI = 0.909, CFI = 0.913, *p* = 0.24) and the Measurement weights path weights change (χ^2^/df = 2.55, RMSEA = 0.39, IFI = 0.913, TLI = 0.911, CFI = 0.913, *p* = 0.004). Interestingly, these weighting changes are also entirely due to the second group (level-2). For example, on the path from emotional self-control to mastery, the second group (*β* = 0.014, *p* < 0.786) was not significant, while the other groups were significant (*β* = 0.142, *p* < 0.011). More interestingly, this group showed a negative and significant correlation from Family recognition through achievement to family emotional support goals (*β* = –0.16, *p* < 0.016), which is not consistent with other groups. It seemed possible to establish a correspondence between level 2 in the highest parental qualification and level 2 in family resources, but there was no consistency between the two questions in terms of results (α = 0.414).

## Discussion

### Cultural values and mathematics goal orientations

The results of our study indicate a significant relationship between cultural values and mathematics learning goal orientation of students in vocational school, which confirms the ideas proposed by previous researchers (Hau and Ho, 2008; Liem et al., 2011; Zusho and Clayton, 2011).

Collectivism is a prominent element of Asian culture (Hau and Ho, 2008; Leung, 2021). Consistent with expectations, collectivism is positively related to family support orientation in mathematics learning. This family support orientation was significant for both instrumental support orientation and family emotional support orientation. This is consistent with the findings of Guo et al. (2021) and Tao and Hong (2014). More importantly, family goal orientation has a higher weighting of emotional support goals than instrumental support orientation, which can be considered collectivism as a more emotionally biased side, i.e., the expectations that Chinese parents impart to their children are more often transformed by the latter into intrinsic motivation and spiritual pursuits. In addition, collectivism and mastery, and performance-approach orientation both have significant positive relationships. This is in line with the assertion of Hau and Ho (2008) that students have the capacity and characteristic to study harder when they are imbibed with collectivism.

This study found a significant relationship between family recognition through achievement and students’ mathematics goal orientation in vocational institutions. First, students’ adherence to this value was associated with stronger mastery, achievement orientation, and performance-avoidance goal orientation. Influenced by Confucianism, Chinese people attach great importance to family reputation in social activities. If Chinese students perceive their achievements as family reputation (Han, 2016), they may strive to gain face for their family members by improving their mathematical ability and outperforming peers in mathematical examinations. On the other hand, they may be concerned that academic failure will bring shame to their families, and thus try to avoid poor performance in math. This study also found that family recognition through achievement did not positively predict students’ family support goal orientation. This finding is contrary to Guo et al. (2021). This may be related to the students’ family background as students in vocational institutions mainly study technology and are not as enthusiastic about learning mathematics as students in ordinary primary and secondary schools, which leads to a disconnect in the goal of gaining family support through learning mathematics.

After we improved the ‘humility’ dimension of the questionnaire, the feedback was very favorable. Humility was found to be a positive predictor of performance-approach orientation and positively predictive with family support goals. This result is consistent with many Western studies (Porter et al., 2020; Krumrei-Mancuso et al., 2020). This is not consistent with the results of Guo et al. (2021). Based on basic discussions with teachers in vocational colleges and universities, it is reasonable to believe that Kim et al.’s (2005) questionnaire has some difficulties in understanding the Chinese context of the dimension of “humility” due to the double negatives in some of the questions, which do not allow students to understand the meaning of the questions well. In addition, “humility” is the only cultural value dimension that has a direct positive correlation with mathematical achievement. It can be said that “humility” enables students to overcome complacency and maintain their motivation to learn (Li, 2016). Specifically, the trait of “modesty” in Chinese culture will encourage vocational school students to set higher learning goals for themselves and constantly yearn for progress in their hearts. This is also an effective confirmation of the relationship between the group’s cultural values and learning goals.

Emotional self-control helps students master what they learn (Graziano et al., 2007). The math proficiency of students in vocational colleges and universities is relatively low, and even fewer are able to overcome the difficulties and persist in learning math content, so being able to control their own emotions will help them better master the content.

Conformity to norms has a negative predictive effect on performance-approach orientation. This result is inconsistent with Guo et al. (2021), but consistent with Spears’s (2021). A plausible explanation is that students want to fit in and be liked by those around them. In order to fit in, participants will change their social identity to adapt to the needs of the group around them. Obedience to norms may have significant negative consequences (Chopot, 2024). Especially, in the campus environment and culture where students in Chinese vocational schools are located, other students may not attach much importance to mathematics learning.

In summary, the five-dimensional framework effectively measures cultural values in the Asian region. Traditional Confucianism contains numerous statements regarding the goal-oriented nature of human learning, such as, “*A gentleman is slow to speak but quick to act*” (*Confucius: The Analects*), and “*Self-restraint and the restoration of propriety constitute benevolence*” (*Confucius: The Analects*). “*To ordain conscience for Heaven and Earth, to secure life and fortune for the populace, to carry on the lost teachings of ancient sages, to build peace for posterity*” (*Zhang Zai: Heng Qu Yi Shuo*). All these discussions will establish meeting the goals of family and society as the driving force and ultimate pursuit of learning, conformity to norms, and collectivism—all family and societal requirements—to ensure students to maintain a stable goal-oriented mindset (Guo et al., 2024). This close association between family and personal success is not unique to Confucianism; similar or analogous findings have been reported in broader cultural contexts (Adamus et al., 2023; Antoniou et al., 2022; Canevello and Crocker, 2020).

### Goal orientations and mathematics achievement

Consistent with previous findings on goal theory (Chen, 2015; Guo et al., 2021), the present study demonstrated that mastery goal orientation positively predicted the mathematics achievement of students in these vocational institutions. One possible reason is that students with high mastery orientation have more behavioral and cognitive engagement in the learning process (King and McInerney, 2019; Wang et al., 2018), which contributes to their success in mathematics.

The present study showed that performance avoidance orientation was negatively related to the mathematics achievement of students in these vocational institutions, which aligns with the finding in previous studies conducted in China (Chen, 2015; Guo et al., 2021). This may be due to the fact that students with high performance avoidance tendencies must devote attentional resources to cope with anxiety and concerns about failure, which reduces their cognitive engagement and self-regulation, which in turn results in poor math performance.

Like Guo et al. (2021), no significant relationship was found between achievement orientation and math achievement, which is inconsistent with the results of some Chinese studies (Chen, 2015). There is reason to believe this is the reason for the scale. We speculate that this might also be related to the weak learning foundation of vocational school students. That is, although they have a strong interest in learning, the weak learning foundation makes it difficult for them to improve their academic performance in a short period of time. King and McInerney (2019) found that the relationship between achievement-oriented goals and student engagement and achievement was not significant when both achievement goals and family support goals were assessed.

It is worth exploring that this does not predict math scores after refining family support goals. This is a significant inconsistency with previous research (King and McInerney, 2019; Guo et al., 2021). A plausible explanation is that math achievement does not figure prominently in the development of students in vocational institutions. This issue was confirmed during interviews with teachers of vocational institutions. Students place more importance on their performance in the professional studies as the proficiency directly determines their future employment, whereas mathematics does not receive sufficient attention. Similarly, for students entering vocational schools, their families themselves do not have expectations for their math grades, which is also difficult to be transformed into a factor for improving their academic performance.

### Goal orientation as a mediator between cultural values and mathematics achievement

This study empirically verified previous inferences that cultural values are closely related to Chinese students’ mathematics achievement (Leung, 2014; Guo et al., 2021) and further explored the potential mechanisms of cultural influence by examining the mediating role of goal orientation. The results indicate that family recognition through achievement influences mathematics achievement through two goal-oriented pathways. Specifically, through student’s mastery, it is positively correlated with mathematics achievement. Family recognition through achievement is negatively correlated with performance avoidance, while performance avoidance is negatively correlated with mathematics achievement. It can be seen that family recognition through achievement is ultimately positively correlated with mathematical achievement in both pathways. These findings align with the double-edged sword model proposed by Peng et al. (2023), who emphasize that parental involvement can simultaneously serve as an empowering force—enhancing motivation and engagement—and as a burden when perceived as stressful. In the context of this study, this can be interpreted as mastery pathways reflecting the empowering aspect, while reduced avoidance behavior illustrating how supportive cognition offsets the burden aspect. Additionally, Wang and Wei (2024) conducted a meta-analysis on parental involvement and math performance, confirming that family support has a significant positive impact, but its effectiveness depends on the type and quality of involvement. The findings of this study provide a more detailed supplement to this discovery: parental recognition is most effective in promoting goal formation and reducing avoidance tendencies. This not only reinforces the positive aspects of the “double-edged sword effect” but also maximizes the benefits students can gain from parental involvement while avoiding potential risks of loss. However, it is also important to be cautious about excessive parental involvement (Deng, 2022), as this may lead to excessive pressure, turning family support into a burden (Gu et al., 2024). Furthermore, this sense of identity should not be overlooked due to family attributes nor ignored due to social group attributes. For example, the influence of ethnic and racial identity depends on the meaning attributed to group membership (Miller-Cotto and Byrnes, 2016), a phenomenon that is particularly pronounced in Asian cultural contexts. Individual students not only face internal group pressures but also undergo socialization comparisons between groups (Abrams et al., 2018). This leads students to develop different identity formations in various contexts, providing a reasonable explanation for the cognitive differences observed in their goal-oriented behaviors. Humility was positively correlated through performance positivity as well as family support orientation and showed a direct positive correlation with math achievement. In addition, the research results indicate that there is a correlation between conformity to norms and the adoption of a mastery orientation. Emotional self-control is positively correlated with students’ mathematics achievements through the mediating variable of mastery orientation. At the same time, the study also found that cultural values are influenced to a certain extent by goal orientation, and among them, the cultural value dimension of “humility” has a direct impact on mathematics achievements.

### Control variables for model impact

The study conducted a multicohort analysis of gender, highest parental education, and family resources, and the results showed no effect on the model, for the weights to change. In this case, gender had a significant impact on the weights. This result has been confirmed in many studies, e.g., PISA (Organization for Economic Cooperation and Development (OECD), 2019). There were significant differences between men and women in pathways, for instance, norm compliance was positively correlated with mastery in math learning, achievement orientation and family instrumental support, and family emotional support orientation. Whereas these paths were not significant in males. The female group is more consistent with Liem and Nie’s (2008) finding that Chinese students’ conformity values positively predicted their mastery goals and socially oriented achievement motivation. A possible explanation for this is that the female group is more norm compliant and that females have a lower mathematical self-concept (Wang, 2019) and may tend to pursue high grades and academic achievement for social recognition, whereas the male group is almost unaffected by this conceptualization.

This situation is the same case with emotional self-control. Family support goal orientation is stronger in the female group, whereas in the male group this linearity is not evident. One possible explanation is that the female group tend to repress their emotions more and have a stronger desire to bring more value to their families, which is in line with the previous study by Wang (2019).

Some interesting phenomena emerged in the grouping results for parents’ highest level of education and family resources. Specifically, the path weights for the second group in the middle showed significant differences from those of the first, third, and fourth groups on certain paths. For example, in the group with second-tier family resources, the path from conformity to norms to family emotional support goal orientation was not significant, whereas the paths were significant in the other three groups. A reasonable explanation is that once family resources reach a certain level, students’ attainment of family emotional support and goal orientation no longer depends on conformity to norms. In fact, as family resources increase, parents may become more reliant on obtaining resources but reduce communication with their children (Xu and Jin, 2024).

## Conclusion

This study explored the relationship between cultural values, goal orientation, family support orientation, and mathematics achievement of 1,004 Liaoning higher vocational college students, mainly from the Han Chinese ethnic group. Our findings confirmed that cultural values play an important role in these students’ mathematics learning, which empirically supports the inferences of previous researchers (Leung, 2014; Wong, 2004; Guo et al., 2021). The present study also verified the strong relationship between goal orientation (including segmented family support goal orientation) and mathematics achievement of these students and obtained different results from previous small samples Guo et al. (2021). In addition, the present study confirmed the mediating role of goal orientation in this relationship through bootstrap analyses, thereby elucidating the underlying mechanism of the relationship between cultural values and mathematics achievement of these students. Finally, this study also analyzed the impact of factors including gender, SES, and other factors on constructing such models.

### Limitations and future work

The study was conducted on the basis of Guo et al. (2021) and verified the significance of many of these relationships, which makes the article slightly less innovative. Secondly, the results of the article do not fully reflect the group qualities of the female side of the group due to the imbalance of the male to female gender ratio in the group of vocational college students. Furthermore, since all data was self-reported and collected via WeChat links, there may be methodological biases in the data, and the results should be treated with caution.

## Data Availability

The raw data supporting the conclusions of this article will be made available by the authors, without undue reservation.
