# Supplementary material for: Culture, goal orientation and achievement of vocational college students
Source: Front Psychol. 2025 Aug 22;16:1639938. doi: 10.3389/fpsyg.2025.1639938 (PMC12412221; doi:10.3389/fpsyg.2025.1639938)
Supplement: Supplementary file 1 [file Data_Sheet_1.PDF]

Cultural and goal oriented data reporting of vocational college students

Sample Description

Table1. Sample Description (N=1004)

		Frequency	Percentage
Gender	Male	887	88.30%
	Female	117	11.70%
	16	1	0.10%
	17	4	0.40%
	18	121	12.10%
	19	345	34.40%
	20	381	37.90%
	21	136	13.50%
	22	10	1%
	23	4	0.40%
	24	1	0.10%
	25	1	0.10%
Parents' highest education level	middle school	396	39.40%
	High school or middle vocational school	307	30.60%
	high vocational school (Associate Degree)	197	19.60%
	Bachelor or Higher	104	10.40%
Books	less than 10	204	20.30%
	10-50	361	36%
	50-100	178	17.70%
	over 100	261	26%

Reliability Analysis

Table2. Reliability Analysis of Cultural Scale and Goal Orientation cale

Reliability analysis of cultural scale and goal orientation scale		
	Cronbach's alpha	items
Norms conformity to norms	0.901	3
SELF emotional self-control	0.866	3
FRA family recognition through achievement	0.857	3
HUM humility	0.926	3
COM collectivism	0.921	3
Culture	**0.946**	**15**
MAS students' mastery	0.920	3

PAP<br>performance-approach,	0.896	3
PAV<br>performance-avoidance	0.863	3
FS<br>family instrumental support goals	0.965	3
FE<br>family emotional support goals	0.957	3
Goal	**0.875**	**15**

Validity

Table3.1 CFA of Cultural Scale

Cultural scale		
Index	Reference indicators	Value
CINM/DF	1-3 Good，3-5 Acceptable	**2.730**
RMSEA	<0.05 Good, <0.08 Acceptable	**0.042**
IFI	>0.9 Good, >0.8 Acceptable	**0.989**
TLI	>0.9 Good, >0.8 Acceptable	**0.985**
CFI	>0.9 Good, >0.8 Acceptable	**0.985**
RFI	>0.9 Good, >0.8 Acceptable	**0.977**
GFI	>0.9 Good, >0.8 Acceptable	**0.971**

Table3.2 Convergence Validity and Combination Reliability of Various Dimensions of Cultural Scale

Path			Estimate	AVE	CR
Norms1	<---	NORM	0.821		
Norms2	<---	NORM	0.905	0.760	0.903
Norms3	<---	NORM	0.882		
Self1	<---	SELF	0.815		
Self2	<---	SELF	0.882	0.690	0.870
Self3	<---	SELF	0.793		
FRA1	<---	FRA	0.823		
FRA2	<---	FRA	0.849	0.667	0.857
FRA3	<---	FRA	0.777		
Hum1	<---	HUM	0.923		
Hum2	<---	HUM	0.897	0.808	0.927
Hum3	<---	HUM	0.876		
Com1	<---	COM	0.880		
Com2	<---	COM	0.905	0.796	0.921
Com3	<---	COM	0.892		

Table3.3 Validity Testing of Differences in Various Dimensions of Cultural Scale

Variables	NORM	SELF	FRA	HUM	COM
NORM	**0.760**				
SELF	0.813	**0.690**			
FRA	0.743	0.780	**0.667**		
HUM	0.560	0.607	0.588	**0.808**	
COM	0.595	0.647	0.611	0.836	**0.796**
Sqrt of AVE	**0.872**	**0.831**	**0.817**	**0.899**	**0.892**

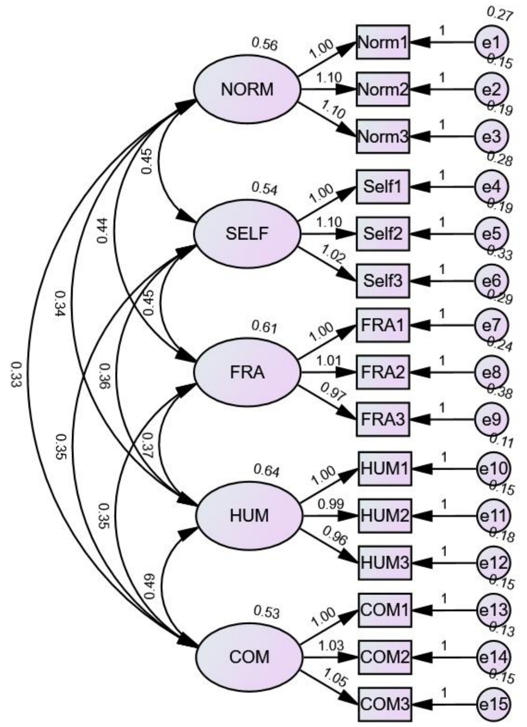

Figure1. Confirmatory Factor Analysis Model of Cultural Scale

Table3.4 CFA of Goal Orientation Scale

Cultural scale		
Index	Reference indicators	Value
CINM/DF	1-3 Good，3-5 Acceptable	**2.620**
RMSEA	<0.05 Good, <0.08 Acceptable	**0.040**
IFI	>0.9 Good, >0.8 Acceptable	**0.991**
TLI	>0.9 Good, >0.8 Acceptable	**0.989**
CFI	>0.9 Good, >0.8 Acceptable	**0.991**
RFI	>0.9 Good, >0.8 Acceptable	**0.982**
GFI	>0.9 Good, >0.8 Acceptable	**0.973**

Table3.5 Convergence Validity and Combination Reliability of Various Dimensions of Goal Orientation Scale

Path			Estimate	AVE	CR
MAS3	<---	MAS	0.90		
MAS2	<---	MAS	0.908	0.793	0.920
MAS1	<---	MAS	0.863		
PAP3	<---	PAP	0.878		
PAP2	<---	PAP	0.864	0.744	0.897
PAP1	<---	PAP	0.845		
PAV3	<---	PAV	0.759		
PAV2	<---	PAV	0.903	0.685	0.866
PAV1	<---	PAV	0.814		
FS3	<---	FS	0.942		
FS2	<---	FS	0.947	0.903	0.965
FS1	<---	FS	0.961		
FE3	<---	FE	0.932		
FE2	<---	FE	0.939	0.883	0.958
FE1	<---	FE	0.948		

Table3.6 Validity Testing of Differences in Various Dimensions of Goal Orientation Scale

Variables	MAS	PAP	PAV	FS	FE
MAS	**0.793**				
PAP	0.791	**0.744**			
PAV	-0.464	-0.399	**0.685**		
FS	0.572	0.658	-0.338	**0.903**	
FE	0.585	0.667	-0.319	0.809	**0.883**
Sqrt of AVE	**0.891**	**0.863**	**0.828**	**0.950**	**0.940**

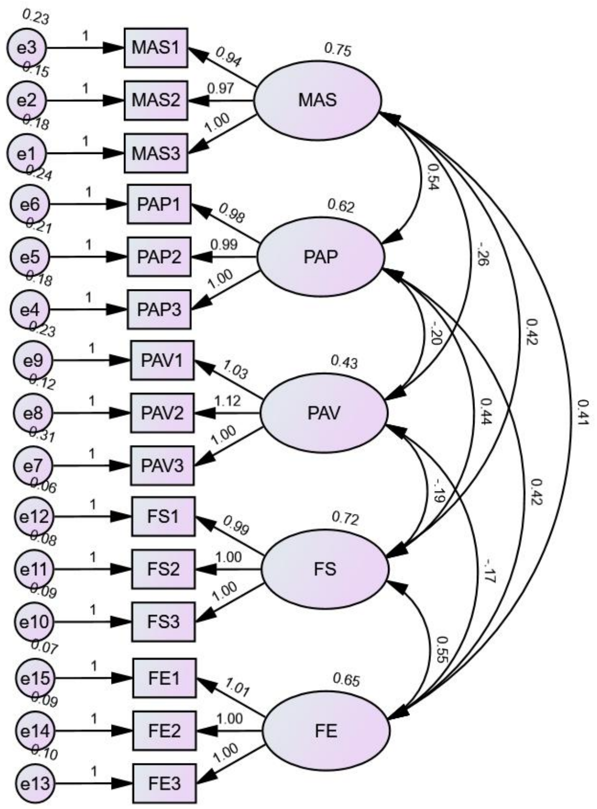

Figure2. Confirmatory Factor Analysis Model of Goal Orientation Scale

Descriptive Statistic and Normality Test

Table4. Normality test of measurement items

		Mean	Std.	S.	K.	Po.Mean	Po.Std.	Po.S.	Po.K.
	Norm1	3.82	0.91	0.04	-1.02				
NORM	Norm2	4.02	0.91	-0.33	-0.91	3.93	0.84	-0.19	-0.76
	Norm3	3.94	0.93	-0.23	-0.94				
	Self1	3.75	0.91	0.09	-0.83				
SELF	Self2	3.86	0.92	-0.12	-0.79	3.77	0.82	0.01	-0.51
	Self3	3.70	0.95	0.05	-0.70				
	FRA1	3.70	0.95	-0.05	-0.53				
FRA	FRA2	3.70	0.94	-0.01	-0.48	3.66	0.84	0.18	-0.32
	FRA3	3.57	0.98	0.01	-0.35				
	HUM1	3.69	0.87	0.16	-0.48				
HUM	HUM2	3.70	0.89	0.11	-0.64	3.71	0.82	0.08	-0.47
	HUM3	3.74	0.88	0.01	-0.61				
	COM1	3.63	0.83	0.26	-0.42				
COM	COM2	3.63	0.83	0.29	-0.45	3.63	0.78	0.29	-0.41
	COM3	3.62	0.86	0.31	-0.48				
	MAS1	3.61	0.94	0.04	-0.33				
MAS	MAS2	3.64	0.93	0.09	-0.43	3.60	0.88	0.24	-0.36
	MAS3	3.56	0.96	0.09	-0.29				
PAP	PAP1	3.53	0.92	0.19	-0.16	3.49	0.82	0.45	0.10

	PAP2	3.43	0.90	0.26	0.23				
	PAP3	3.52	0.90	0.22	-0.02				
	PAV1	2.65	0.83	-0.50	0.50				
PAV	PAV2	2.60	0.81	-0.69	0.21	2.59	0.74	-0.93	0.12
	PAV3	2.52	0.86	-0.46	-0.22				
	FS1	3.64	0.88	0.19	-0.31				
FS	FS2	3.65	0.90	0.14	-0.37	3.64	0.86	0.20	-0.28
	FS3	3.64	0.90	0.17	-0.38				
	FE1	3.61	0.86	0.31	-0.35				
FE	FE2	3.62	0.86	0.29	-0.41	3.61	0.83	0.36	-0.27
	FE3	3.60	0.87	0.33	-0.39				

Pearson Correlation

Table5. Pearson Correlation

	AVENorm	AVESelf	AVEFRA	AVEHUM	AVECOM	AVEMAS	AVEPAP	AVEPAV	AVEFS	AVEFE
AVENorm	1									
AVESelf	.725**	1								
AVEFRA	.665**	.680**	1							
AVEHUM	.526**	.552**	.524**	1						
AVECOM	.553**	.586**	.539**	.771**	1					
AVEMAS	.648**	.687**	.686**	.547**	.603**	1				
AVEPAP	.558**	.611**	.685**	.605**	.637**	.717**	1			
AVEPAV	-.377**	-.395**	-.373**	-.328**	-.339**	-.421**	-.356**	1		
AVEFS	.524**	.551**	.523**	.849**	.756**	.540**	.613**	-.310**	1	
AVEFE	.536**	.548**	.501**	.772**	.825**	.551**	.620**	-.285**	.778**	1

SEM

Table6.1 SEM of Culture, Goal orientation, and Math Achievement

Cultural scale		
Index	Reference indicators	Value
CINM/DF	1-3 Good，3-5 Acceptable	**3.229**
RMSEA	<0.05 Good, <0.08 Acceptable	**0.047**
IFI	>0.9 Good, >0.8 Acceptable	**0.972**
TLI	>0.9 Good, >0.8 Acceptable	**0.966**
CFI	>0.9 Good, >0.8 Acceptable	**0.972**
RFI	>0.9 Good, >0.8 Acceptable	**0.952**
GFI	>0.9 Good, >0.8 Acceptable	**0.923**

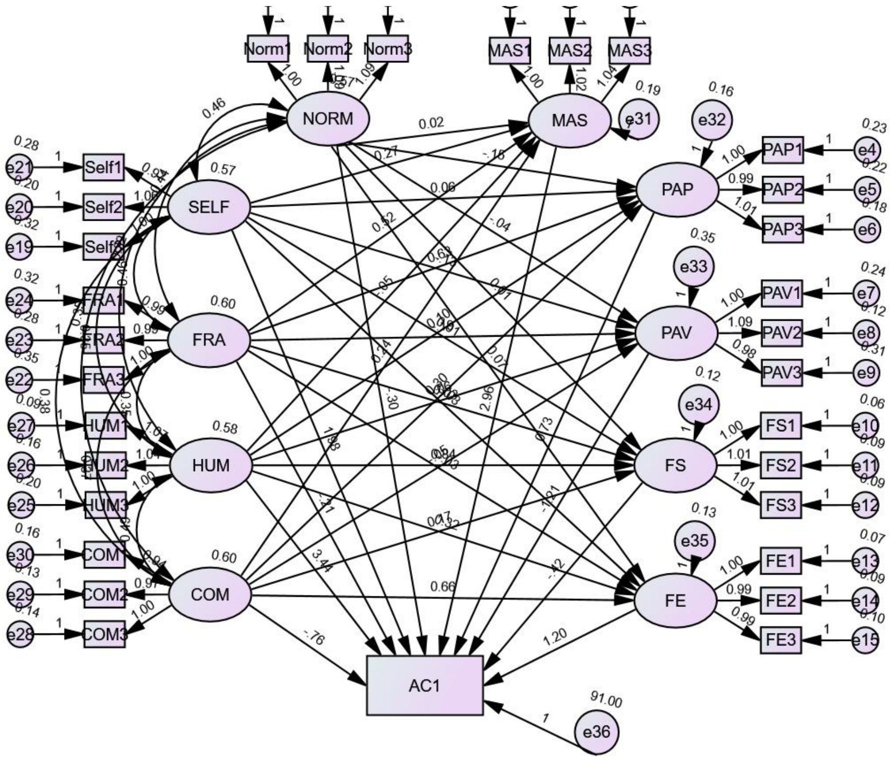

Figure3. SEM of Culture, Goal orientation and Math Achievement

Table6.2 Path Estimation in SEM

Path			S.t.Estimate	P
MAS	<---	NORM	0.019	0.685
PAP	<---	NORM	-0.143	0.003
PAV	<---	NORM	-0.045	0.506
FS	<---	NORM	0.01	0.776
FE	<---	NORM	0.068	0.065
MAS	<---	SELF	0.246	***
PAP	<---	SELF	0.059	0.301
PAV	<---	SELF	-0.148	0.063
FS	<---	SELF	0.007	0.863
FE	<---	SELF	-0.028	0.526
MAS	<---	FRA	0.487	***
PAP	<---	FRA	0.625	***
PAV	<---	FRA	-0.222	***
FS	<---	FRA	0.023	0.492
FE	<---	FRA	-0.03	0.402
MAS	<---	HUM	-0.047	0.293
PAP	<---	HUM	0.094	0.044
PAV	<---	HUM	-0.07	0.286
FS	<---	HUM	0.758	***
FE	<---	HUM	0.303	***
MAS	<---	COM	0.227	***
PAP	<---	COM	0.302	***

PAV	<---	COM	-0.058	0.402
FS	<---	COM	0.152	***
FE	<---	COM	0.876	***
AC1	<---	NORM	-0.02	0.752
AC1	<---	SELF	0.131	0.071
AC1	<---	FRA	-0.014	0.875
AC1	<---	HUM	0.228	0.015
AC1	<---	COM	-0.051	0.557
AC1	<---	MAS	0.213	***
AC1	<---	PAP	0.05	0.462
AC1	<---	PAV	-0.071	0.031
AC1	<---	FS	-0.031	0.696
AC1	<---	FE	0.085	0.24

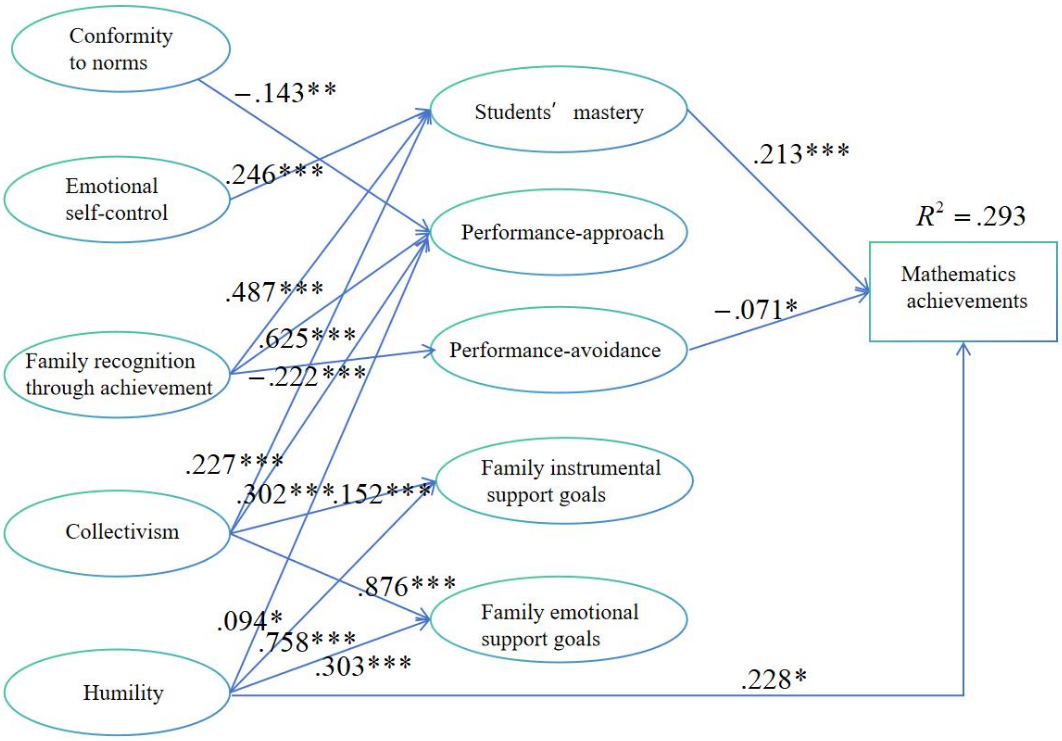

Figure4. Significant path details ($*p<.05$, $**p<.01$, $***p<.001$)

Table6.3. Mediation Effect Test of Bootstrap 5000 (95%)

Parameter			Indirect Effects	Path coefficients	95%Confidence Intervals	P
MAS	<---	FRA	.130	.487	[.287,.785]	***
PAP	<---	FRA	.151	.625	[.410,.973]	***
PAV	<---	FRA	.105	-.222	[-.439,-.022]	**
FE	<---	HUM	.061	.303	[.190,.433]	***
MAS	<---	COM	.075	.227	[.077,.371]	**
PAP	<---	COM	.081	.302	[.141,.459]	***
FS	<---	COM	.061	.152	[.041,.278]	**
FE	<---	COM	.068	.626	[.475,.750]	***
Achievement	<---	HUM	.054	.228	[.122,.337]	*
Achievement	<---	MAS	.063	.213	[.093,.336]	**
Achievement	<---	PAV	.032	-.071	[-.138,-.012]	*

(*p<.05,**p<.01,***p<.001)

Multiple-Group Analysis

Table7.1 Gender

Model	CMIN	DF	CMIN/DF	RSME	RFI	IFI	TLI	CFI
Unconstrained	1873.446	780	**2.402**	0.037	0.931	0.965	0.958	0.965
Measurement weights	1910.268	810	**2.358**	0.037	0.932	0.965	0.96	0.965
Structural weights	1966.913	835	**2.356**	0.037	0.932	0.964	0.96	0.964
Structural covariances	1995.248	850	**2.347**	0.037	0.932	0.963	0.96	0.963
Structural residuals	1999.395	855	**2.338**	0.037	0.932	0.963	0.96	0.963

Table 7.2 Assuming Model Unconstrained to be correct

Model	DF	CMIN	P	IFI	RFI	TLI
Measurement weights	30	36.822	**0.182**	0.001	-0.001	-0.001
Structural weights	55	93.467	0.001	0.003	-0.001	-0.001
Structural covariances	70	121.803	0	0.004	-0.002	-0.002

Structural residuals	75	125.95	0	0.004	-0.002	-0.002

Table7.3 Assuming model Measurement weights to be correct

Model	DF	CMIN	P	IFI	RFI	TLI
Structural weights	25	56.645	**0**	0.002	0	0
Structural covariances	40	84.98	0	0.003	0	0
Structural residuals	45	89.128	0	0.003	-0.001	-0.001

Table7.4 Impact of Gender on Different Pathways

	Path		Estimate	P	Estimate	P
MAS	<---	NORM	-0.032	0.531	0.398	0.005
PAP	<---	NORM	-0.215	***	0.41	0.009
FS	<---	NORM	0.012	0.742	0.271	0.019
FE	<---	NORM	0.053	0.191	0.395	***
MAS	<---	SELF	0.269	***	-0.203	0.275
FS	<---	SELF	0.045	0.313	-0.525	0.001
FE	<---	SELF	0.003	0.944	-0.62	***
MAS	<---	FRA	0.506	***	0.596	***
PAP	<---	FRA	0.653	***	0.661	***
PAV	<---	FRA	-0.227	0.003	-0.084	0.572
FS	<---	FRA	-0.024	0.528	0.291	***
PAP	<---	HUM	0.1	0.04	-0.01	0.953
FS	<---	HUM	0.785	***	0.521	***
FE	<---	HUM	0.331	***	-0.147	0.247
MAS	<---	COM	0.213	***	0.433	0.011
PAP	<---	COM	0.301	***	0.333	0.081
FS	<---	COM	0.13	***	0.47	***
FE	<---	COM	0.593	***	1.226	***
AC1	<---	HUM	0.219	0.034	0.106	0.693
AC1	<---	MAS	0.203	0.002	0.151	0.481
AC1	<---	PAV	-0.08	0.023	0.082	0.357

Table7.5 Impact of Gender on Different Pathways (Bootstrap 5000,95%)

Parameter			Male				Female			
			Estimate	Lower	Upper	P	Estimate	Lower	Upper	P
PAP	<---	NORM	-0.22	-0.412	-0.031	**	0.468	-0.034	4.215	0.07
FE	<---	NORM	0.057	-0.036	0.161	0.215	0.474	0.028	8.597	**
FE	<---	SELF	0.004	-0.152	0.149	0.922	-0.681	-7.761	-0.065	**
MAS	<---	FRA	0.543	0.25	1	*	0.572	0.329	3.101	**
PAP	<---	FRA	0.656	0.367	1.131	**	0.669	0.2	2.441	**
FS	<---	FRA	-0.026	-0.146	0.128	0.701	0.339	0.024	2.388	*
FS	<---	HUM	0.861	0.689	1.019	***	0.629	-2.414	1.311	0.54
FE	<---	HUM	0.354	0.223	0.516	***	-0.162	-6.079	0.365	0.373
MAS	<---	COM	0.231	0.061	0.406	*	0.383	-0.214	2.385	0.127
PAP	<---	COM	0.306	0.139	0.473	**	0.311	-0.573	1.79	0.371
FS	<---	COM	0.141	0.017	0.289	*	0.504	-0.113	2.741	0.091
FE	<---	COM	0.628	0.462	0.778	***	1.205	0.702	6.021	*
AC1	<---	HUM	3.354	0.289	6.206	*	1.268	-47.642	87.428	0.775
AC1	<---	MAS	2.849	0.884	4.609	**	1.82	-5.567	18.123	0.485
AC1	<---	PAV	-1.393	-2.722	-0.274	*	1.02	-1.673	3.875	0.453

Table7.6 Parents' highest education level

Model	CMIN	DF	CMIN/DF	RMSEA	RFI	IFI	TLI	CFI
**Unconstrained**	4557.523	1772	**2.572**	0.04	0.862	0.915	0.911	0.915
**Measurement weights**	4596.485	1802	**2.551**	0.039	0.863	0.915	0.912	0.915
**Structural weights**	4648.558	1827	**2.544**	0.039	0.863	0.914	0.912	0.914
**Structural covariances**	4686.138	1842	**2.544**	0.039	0.863	0.913	0.912	0.913
**Structural residuals**	4699.832	1847	**2.545**	0.039	0.863	0.913	0.912	0.913

Table 7.7 Assuming Model Unconstrained to be correct

Model	DF	CMIN	P	IFI	RFI	TLI
Measurement weights	30	38.961	**0.127**	0.001	0.001	-0.001
Structural weights	55	91.035	0.002	0.003	0.003	-0.001
Structural covariances	70	128.615	0	0.004	0.004	-0.001
Structural residuals	75	142.309	0	0.004	0.004	-0.001

Table7.8 Assuming model Measurement weights to be correct

Model	DF	CMIN	P	IFI	RFI	TLI
Structural weights	25	52.073	0.001	0.002	0.002	0
Structural covariances	40	89.654	0	0.003	0.003	0
Structural residuals	45	103.347	0	0.003	0.003	0

Table7.9 Impact of parental education on the model

			Middle School		High School		High Vocation		Bachelar	
	Path		Estimate	P	Estimate	P	Estimate	P	Estimate	P
MAS	<---	SELF	0.221	***	0.473	**0.022**	0.221	***	0.221	***
MAS	<---	FRA	0.427	***	0.582	***	0.427	***	0.427	***
PAP	<---	FRA	0.601	***	0.544	***	0.601	***	0.601	***
FS	<---	HUM	0.784	***	0.962	***	0.784	***	0.784	***
FE	<---	HUM	0.29	***	0.401	***	0.29	***	0.29	***
MAS	<---	COM	0.237	***	0.185	**0.034**	0.237	***	0.237	***
PAP	<---	COM	0.312	***	0.251	**0.003**	0.312	***	0.312	***
FS	<---	COM	0.167	***	0.145	**0.032**	0.167	***	0.167	***
FE	<---	COM	0.662	***	0.631	***	0.662	***	0.662	***
AC1	<---	MAS	2.784	***	2.784	***	2.784	***	2.784	***

Table7.10 Family Resources and Models

Model	CMIN	DF	CMIN/DF	RMSEA	RFI	IFI	TLI	CFI
**Unconstrained**	4559.332	1772	**2.573**	0.04	0.86	0.914	0.909	0.913
**Measurement weights**	4594.398	1802	**2.55**	0.039	0.861	0.913	0.911	0.913
**Structural weights**	4642.272	1827	**2.541**	0.039	0.861	0.913	0.911	0.913
**Structural covariances**	4683.955	1842	**2.543**	0.039	0.861	0.912	0.911	0.912
**Structural residuals**	4686.592	1847	**2.537**	0.039	0.862	0.912	0.911	0.912

Table 7.11 Assuming Model Unconstrained to be correct

Model	DF	CMIN	P	IFI	RFI	TLI
Measurement weights	30	35.066	**0.24**	0.001	-0.001	-0.001
Structural weights	55	82.94	0.009	0.003	-0.002	-0.002
Structural covariances	70	124.623	0	0.004	-0.002	-0.002
Structural residuals	75	127.26	0	0.004	-0.002	-0.002

Table7.12 Assuming model Measurement weights to be correct

Model	DF	CMIN	P	IFI	RFI	TLI
Structural weights	25	47.874	0.004	0.001	0	-0.001
Structural covariances	40	89.557	0	0.003	0	0
Structural residuals	45	92.194	0	0.003	-0.001	-0.001

Table7.13 Impact of Family Resources on the model

Path			Level-1		Level-2		Level-3		Level-4	
			Estimate	P	Estimate	P	Estimate	P	Estimate	P
**PAP**	<---	**NORM**	-0.103	0.144	-0.177	0.008	-0.103	0.144	-0.103	0.144
**FE**	<---	**NORM**	0.142	0.011	0.014	0.786	0.142	0.011	0.142	0.011
**MAS**	<---	**SELF**	0.165	0.025	0.318	***	0.165	0.025	0.165	0.025
**FE**	<---	**SELF**	-0.144	0.02	0.094	0.161	-0.144	0.02	-0.144	0.02
**MAS**	<---	**FRA**	0.538	***	0.398	***	0.538	***	0.538	***
**PAP**	<---	**FRA**	0.682	***	0.611	***	0.682	***	0.682	***
**PAV**	<---	**FRA**	-0.281	***	-0.158	0.193	-0.281	***	-0.281	***
**FE**	<---	**FRA**	0.037	0.406	-0.16	0.016	0.037	0.406	0.037	0.406
**PAP**	<---	**HUM**	0.034	0.546	0.174	0.034	0.034	0.546	0.034	0.546
**FS**	<---	**HUM**	0.725	***	0.859	***	0.725	***	0.725	***
**FE**	<---	**HUM**	0.249	***	0.418	***	0.249	***	0.249	***
**MAS**	<---	**COM**	0.235	***	0.244	0.003	0.235	***	0.235	***
**PAP**	<---	**COM**	0.368	***	0.177	0.041	0.368	***	0.368	***
**PAV**	<---	**COM**	-0.18	0.03	0.205	0.094	-0.18	0.03	-0.18	0.03
**FS**	<---	**COM**	0.166	***	0.095	0.172	0.166	***	0.166	***
**FE**	<---	**COM**	0.664	***	0.553	***	0.664	***	0.664	***
**AC1**	<---	**HUM**	0.237	0.012	0.212	0.012	0.237	0.012	0.237	0.012
**AC1**	<---	**MAS**	0.217	***	0.195	***	0.217	***	0.217	***
**AC1**	<---	**PAV**	-0.07	0.036	-0.067	0.036	-0.07	0.036	-0.07	0.036
